# Predictive Indicators for Necrotizing Enterocolitis With the Presence of Portal Venous Gas and Outcomes of Surgical Interventions

**DOI:** 10.3389/fped.2021.683510

**Published:** 2021-06-14

**Authors:** Xin Lin, Hui-Ping Zeng, Yi-Fan Fang, Ying-Ying Lin, Chang-Yi Yang

**Affiliations:** ^1^Department of Neonatology, Fujian Maternity and Child Health Hospital, Affiliated Hospital of Fujian Medical University, Fuzhou, China; ^2^Department of Pediatric Surgery, Fujian Maternity and Child Health Hospital, Affiliated Hospital of Fujian Medical University, Fuzhou, China; ^3^Department of Healthcare, Fujian Maternity and Child Health Hospital, Affiliated Hospital of Fujian Medical University, Fuzhou, China

**Keywords:** predictive indicators, outcomes, necrotizing enterocolitis, portal venous gas, surgical intervention

## Abstract

**Objectives:** Portal venous gas (PVG) was an important clinical sign in stage II or III necrotizing enterocolitis (NEC) in preterm neonates. Not a proper predictive indicator was found to predict the diseases (NEC with the presence of PVG) up to now. There is a need to put forward predictive indicators and compare the predictive effects among them.

**Methods:** We conducted a retrospective study of preterm neonates with NEC-PVG (*n* = 61) or NEC-non PVG (*n* = 62) from 2014 to 2021. Predictive indicators were put forward and determined by receiver operating characteristic curve analysis. An analysis of the surgical interventions and their outcomes was performed.

**Results:** The incidence rate of NEC among preterm neonates was 4.99%; surgical and conservative interventions accounted for 20.47 and 75.07%, and the mortality rate was 0.03%. The composition ratio of shock in the NEC-PVG group increased 13.2% (*P* = 0.029). C-reactive protein, fibrinogen degradation product, and blood glucose had better predictive effects in the predictive indicators (*P* < 0.05). Intestinal necrosis and subependymal hemorrhage in the outcomes of surgical interventions had a strong relationship with the presence of PVG in NEC II/III (*P* < 0.05).

**Conclusion:** Early and reasonable use of antibiotics, improvement of coagulation function, rectification of acidosis, and decreased blood glucose could cut down the occurrence of the disease (NEC with the presence of PVG). Except for subependymal hemorrhage and intestinal necrosis, NEC with the presence of PVG did not increase the occurrence of other outcomes after surgery.

## Introduction

Necrotizing enterocolitis (NEC) is a serious intestinal disease caused by the combined effects of immaturity, infection, ingestion, ischemia injury, insufficient oxygenation, and immunological factors, which threatens the life of neonates (1). In the neonatal intensive care unit (NICU), the incidence rate of NEC is 2–5% overall and 4.5–8.7% among very low birth weight (VLBW) neonates, and the mortality rate is 20–30% (2, 3). The absolute indication for NEC surgery is intestinal perforation (4, 5), while conservative treatment failure and worsening of the condition are also relative indications for surgery (5). Almost 30–50% of neonates with NEC eventually require surgical intervention. The stricture, short bowel syndrome (SBS) and neurodevelopmental impairment are sequelae for NEC (6). Portal venous gas (PVG) was an important clinical sign in stage II or III NEC in preterm neonates. The detection of PVG on abdominal ultrasound (AUS) (as opposed to on X-ray examination) was proven to be as early sign of impending NEC in VLBW neonates (7), and sonographic findings of PVG (OR = 3.9) were significantly associated with surgery and/or death (8). Moreover, PVG was detected by the AUS with high specificity (86–100%) and proper sensitivity (3–45%) in the diagnostic value of NEC in preterm neonates (9). Although the signs of PVG could be detected by X-ray in the severe NEC as well, repeating the examinations of X-ray increased exposure to radiation and did harm to preterm neonates. Thus, X-ray was not chosen as a predictive indicator in the study. Of the mechanism of NEC (10), changes in inflammation indicators were used at the early diagnosis of infections (11, 12); procoagulant status of coagulation function indicators was found at the start of NEC in prematurity (13); abnormity of blood lactate concentrations in the preoperative period carries a poor prognosis in neonates with NEC (14). However, whether these can be used as the predictive indicators on the disease (NEC with the presence of PVG) is still uncertain. There is an urgent need to find predictive indicators with both high sensitivities and specificity and to compare the predictive effects among them.

This retrospective study not only put forward the proper predictive indicators for the disease but also analyzed the outcomes of surgical interventions whether or not they had the relationship with the presence of PVG in NEC II/III.

## Materials and Methods

### Study Population

#### The Criteria of Inclusion and Exclusion

Preterm neonates diagnosed with stage II or III NEC with a gestational age (GA) of 25–36 weeks were included in NICU from January 2014 to January 2021, while neonates diagnosed with stage I were excluded. According to the classification of Bell's criterial (15), stage II NEC was diagnosed as neonates with the following symptoms: ①the systemic symptoms: temperature instability, apnea, irritability, or lethargy; ②the gastrointestinal symptoms: abdominal distension, abdominal mass, blood in stools, or abdominal rumbling sound weakening; ③the results of iconography: intestinal obstruction, gas in the bowel wall, PVG, or ascites. Systemic symptoms (persistent low blood pressure, bradycardia, and shock), intestinal perforation, and acute diffuse peritonitis were added to stage III NEC. Stage I NEC was determined as neonates with blood in stools and mild gastrointestinal symptoms that did not reach the criterial of stage II.

**Figure 1:**
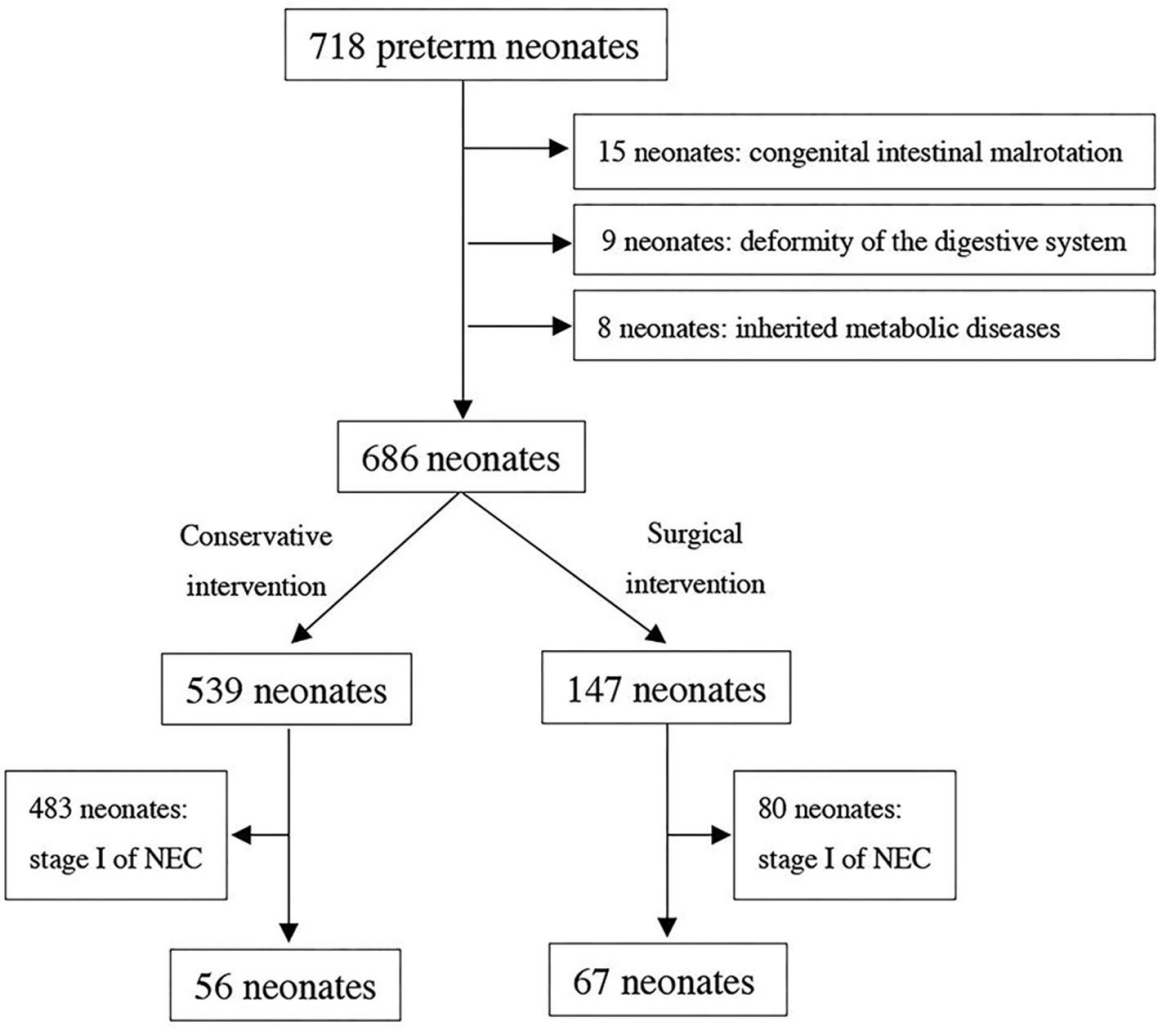
Flow diagram for patient population of necrotizing exterocolitis.

Because of congenital intestinal malrotation, deformity of the digestive system, or inherited metabolic diseases, some preterm neonates among them were excluded. As a result, the included neonates with complete clinical data were divided into two groups according to conservative and surgical interventions, as shown in Figure 1.

#### The Demographic Characteristics

The demographic characteristics of neonates were recorded: the stage of NEC, GA and birth weight (BW), small for gestational age (SGA), male, asphyxia, and last volume of milk. Simultaneously, the characteristics of mothers were recorded: mothers' age, mode of delivery, premature rupture of membranes, turbid amniotic fluid, multiple pregnancies, prenatal use of antibiotics, and main maternal pregnancy diseases (e.g., gestational diabetes mellitus (GDM), intrauterine infection (IAI), hypertension during pregnancy, and anemia in pregnancy).

The characteristics of neonates when NEC happened were recorded, including apnea, temperature instability, shock, ascites, acute peritonitis, and length of stay in hospital. Acute peritonitis was indicated by a triad of peritoneal irritation (tenderness, abdominal muscle tension, and rebound tenderness). Ascites were detected by AUC when NEC occurred. Breathing that stopped for more than 20 s was accompanied by slowing of the heart rate to <100 beats/min, and decreased blood oxygen saturation was diagnosed as apnea. Neonatal shock was defined as changes in skin color, slower skin circulation, decreased limb temperature, weakened femoral artery fluctuations, and decreased blood pressure (16). Temperature instability was diagnosed as rectal temperature lower than 36.0°C or higher than 37.5°C when NEC happened.

### Parameter Variables

#### The Examination of AUS

A total of 123 preterm neonates were divided into two groups: a NEC-PVG group (*n* = 61) and a NEC-non PVG group (*n* = 62). PVG refers to the imaging signs indicating the accumulation of gas in the portal vein and its branches in the liver and can easily be identified under AUS with the low echogenic flat background noise of the ordinary bloodstream (17). All preterm neonates were examined by AUS (Volumson S8, General Electric Healthcare ultrasound) to determine whether they had signs of PVG or ascites, once the suspicious systemic or gastrointestinal symptoms of NEC appeared.

#### The Examination of Laboratory Parameters

Percentage of neutrophils (NEUT%) refers to the percentage of neutrophils in all blood cells. The neutrophil/lymphocyte (N/L) ratio is determined as absolute neutrophil counts (NE) divided by absolute lymphocyte counts (LY). When suspicious symptoms of NEC appeared, routine blood indicators [white blood cells (WBC) count, NEUT%, NE, LY, N/L ratio, platelet count (PLT), and C-reactive protein (CRP)] were measured in venous blood samples (1 ml) using an automatic blood cell analyzer (BC-5390CRP, Mindray Co), and prothrombin time (PT), activated partial thromboplastin time (APTT), D-dimer, and fibrinogen degradation product (FDP) were measured in venous blood samples (2 ml) using an automatic blood coagulation analyzer (CS-5100, SYSMEX Co). Neonates received blood gas analysis (pH value, lactate levels, and blood glucose) with venous blood samples (0.5 ml) using an automatic blood gas analyzer (GEM Premier 4000, Instrumentation Laboratory Co.).

### Surgical Interventions and Outcomes

According to the area of necrosis, whether having intestinal perforations or not and clinical symptoms, four kinds of surgical interventions were carried out by surgeon, including laparotomy, enterostomy, intestinal resection and enterostomy, and intestinal resection and anastomosis.

Intraoperative bowel morphology and postoperative complications were recorded for outcomes in both the NEC-PVG group and NEC-non PVG group among neonates with surgery. Intestinal perforation, bleeding, and necrosis were confirmed through the first intraoperative bowel morphology and postoperative pathological results. Intestinal stricture and obstruction were diagnosed in the second intraoperative bowel morphology or postoperative pathological results. If the remaining small intestine was <50% of the length among neonates of the same age and compounded by malabsorption and disordered motility after surgery, SBS was indicated (18). Subependymal hemorrhage was diagnosed by transcranial ultrasound as a sequela after the first surgery of NEC.

### Statistical Analysis

The statistical analyses were performed using SPSS Statistics version 26.0 (IBM Corp, Armonk, NY, USA). The demographic characteristics were expressed as the mean ± SD (standard deviation) or the median (P25, P75) for continuous data and number (%) for categories variables. In analyses of continuous data, if fit to a normal distribution, the variables were analyzed by *t*-tests; if not, they were analyzed by the Mann–Whitney U test. Chi-square tests or Fisher's exact tests were used in the analyses of categorical variables. A receiver operating characteristic curve (ROC curve) was generated to compare the area under the curve (AUC), sensitivity, specificity, and cutoff values. Results with *P* < 0.05 were considered statistically significant.

## Results

### Study Population

From January 2014 to January 2021, 718 of 14,387 preterm neonates diagnosed with stage II or III NEC with a GA of 25–36 weeks were recorded. After 15 neonates of congenital intestinal malrotation, 9 neonates of deformity of the digestive system, and 8 neonates of inherited metabolic diseases were excluded, 686 preterm neonates were selected primarily. Among them, 147 underwent surgical intervention and 539 underwent conservative intervention. After excluding the neonates diagnosed with stage I NEC (483 neonates of conservative intervention and 80 neonates of surgical intervention), a total of 123 preterm neonates were eventually included in the study, as shown in Figure 1.

In NICU, the incidence rate of NEC among preterm neonates was 4.99% (718/14,387) within 7 years; among these neonates' surgical interventions were performed in 20.47% (147/718) and conservative interventions accounting for 75.07% (539/718). The mortality rate of NEC in preterm neonates was 0.03% (4/14,387). In the NEC-PVG group, the mean GA was 30.5 ± 2.1 weeks, and the mean BW was 1408.7 ± 329.2 g. In the NEC-non PVG group, the mean GA was 29.9 ± 2.2 weeks, and the mean BW was 1350.7 ± 331.9 g. The demographic characteristics of the neonates among two groups were reported in Table 1.

**Table 1:** The demographic characteristics of NEC-PVG group and NEC-non PVG group.

**Variables**	**NEC-PVG**	**NEC-non PVG**	***P***
	**(*n* = 61)**	**(*n* = 62)**	
NEC stage III	30 (49.2%)	21 (33.9%)	0.085
GA (week)	30.5 ± 2.1	29.9 ± 2.2	0.182
BW (g)	1408.7 ± 329.2	1350.7 ± 331.9	0.333
Male	36 (59.0%)	42 (67.7%)	0.315
SGA	12 (19.7%)	8 (12.9%)	0.309
Asphyxia	10 (16.4%)	12 (19.4%)	0.668
Last volume of milk	23 (19, 30)	25 (17, 33)	0.334
Mother's age	30.0 ± 4.6	30.5 ± 5.0	0.591
Vaginal delivery	26 (42.6%)	34 (54.8%)	0.175
Premature rupture of membranes	16 (26.2%)	20 (32.3%)	0.463
Turbid amniotic fluid	12 (19.7%)	14 (22.6%)	0.693
Multiple pregnancy	15 (24.6%)	13 (21.0%)	0.632
GDM	13 (21.3%)	15 (24.2%)	0.703
Hypertension during pregnancy	12 (19.7%)	13 (21.0%)	0.858
Anemia in pregnancy	23 (37.7%)	24 (38.7%)	0.909
IAI	16 (26.2%)	15 (24.2%)	0.795
Apnea	46 (75.4%)	45 (72.6%)	0.721
Temperature instability	26 (42.6%)	28 (45.2%)	0.777
Shock	12 (19.7%)	4 (6.5%)	0.029
Ascites	25 (41.0%)	23 (37.1%)	0.659
Acute peritonitis	31 (50.8%)	11 (17.7%)	<0.01
Length of stay in hospital	48 (33, 59)	46 (37, 59)	0.990

*NEC, necrotizing enterocolitis; GA, gestational age; BW, birth weight; SGA, small for gestational age; GDM, gestational diabetes mellitus; IAI, intrauterine infection*.

The composition ratio of shock in the NEC-PVG group increased 13.2% compared to the NEC-non PVG group (*P* = 0.029). There was significant difference of acute peritonitis between two groups (*P* < 0.01). The composition ratios of apnea, temperature instability, or ascites were similar among two groups (*P* > 0.05). Compared to neonates (46 days) in the NEC-non PVG group, the median length of stay of the NEC-PVG group was 48 days (*P* = 0.990).

### The Predictive Effects of Laboratory Parameters

#### The Inflammatory Indicators

**Figure 2:**
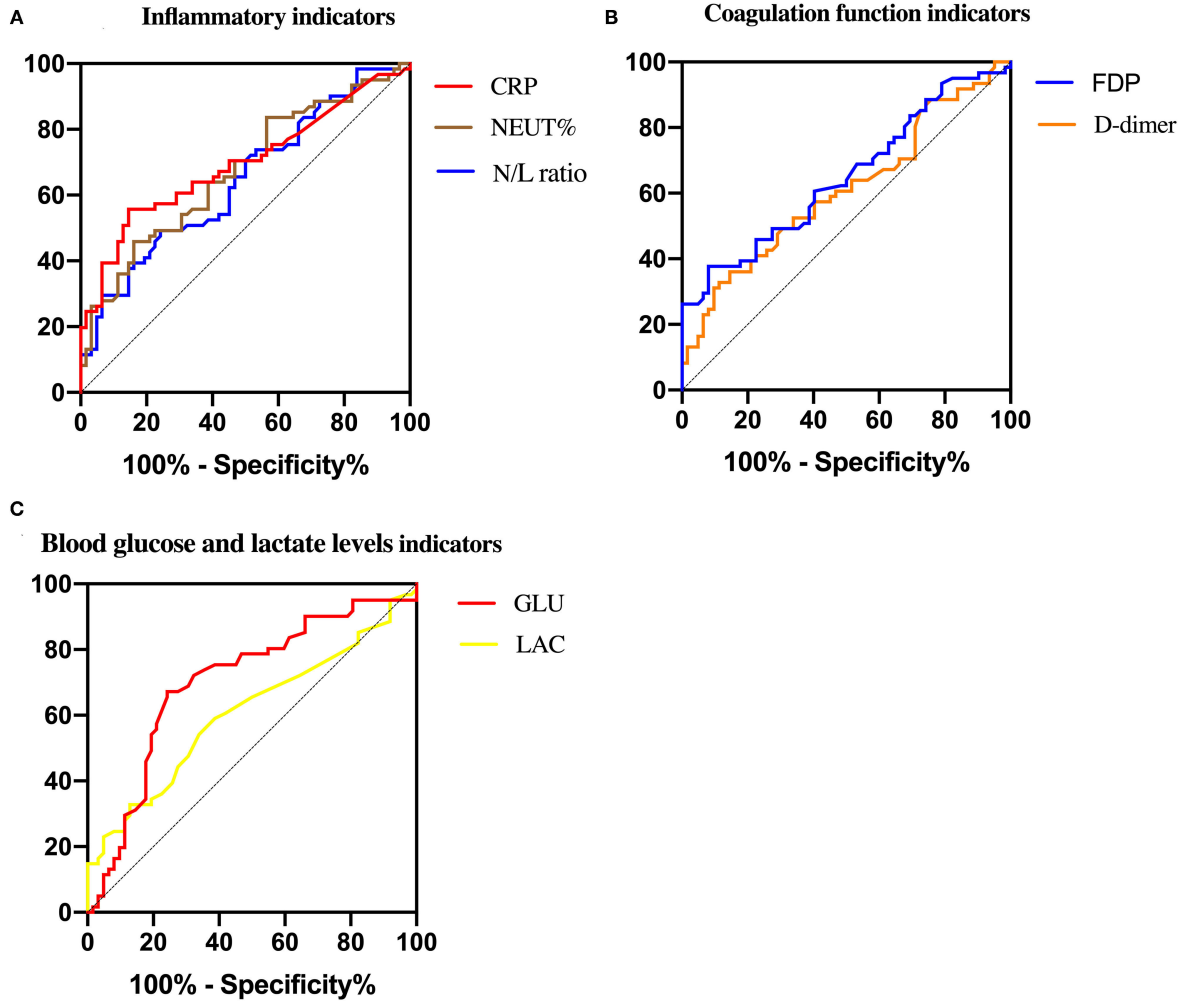
Roc curve of predictive indicators between NEC-PVG group and NEC-non PVG group.

An analysis comparing the inflammatory indicators between the two groups of neonates yielded a significant relationship between NEUT% and the disease (NEC with the presence of PVG) (*P* = 0.001, Table 2). The average value of NEUT% in the NEC-PVG group was much higher than that in the NEC-non PVG group (55.5 ± 18.0 vs. 44.5 ± 16.1), while there was no significant difference in the WBC count between them (*P* = 0.633). The median LY in the NEC-PVG group (1.84 × 10^9^/*L*) was significantly lower than that (2.56 × 10^9^/*L*) in the NEC-non PVG group (*P* = 0.013), while there were no differences of the median NE (*P* = 0.373) between them. The N/L ratio in the NEC-PVG group was significantly higher than that in the NEC-non PVG group (*P* = 0.048, Table 2). CRP was a significantly different between two groups (6.59 vs. 0.82, *P* < 0.01). CRP showed the largest AUC (0.694), and its specificity (0.86) was larger than those for the N/L ratio and NEUT% (shown in Table 3 and Figure 2).

**Table 2:** Laboratory parameters of NEC-PVG group and NEC-non PVG group.

**Variables**	**NEC-PVG**	**NEC-non PVG**	***P***
	**(*n* = 61)**	**(*n* = 62)**	
**Inflammatory indicators**			
WBC count (×10^9^/*L*)	6.87 (4.14, 10.78)	7.42 (5.64, 10.32)	0.633
NEUT (%)	55.50 ± 18.03	44.51 ± 16.08	0.001
NE (×10^9^/*L*)	3.39 (2.18, 5.74)	3.38 (1.75, 4.81)	0.373
LY (×10^9^/*L*)	1.84 (1.04, 2.93)	2.56 (1.50, 3.84)	0.013
N/L ratio	1.79 (1.02, 3.41)	1.24 (0.68, 2.03)	0.048
CRP (mg/L)	6.59 (0.54, 16.80)	0.82 (0.50, 2.47)	<0.01
**Coagulation function indicators**			
PLT (×10^9^/*L*)	258.0 (174.5, 314.5)	216.0 (162.8, 272.5)	0.115
PT (s)	14.80 (13.53, 16.80)	14.45 (13.40, 16.63)	0.557
APTT (s)	57.00 (46.50, 79.55)	53.85 (41.90, 67.65)	0.106
D-dimer (g/L)	1.70 (0.93, 4.45)	1.28 (0.83, 2.14)	0.039
FDP (mg/L)	5.98 (4.24, 12.89)	5.16 (3.12, 6.87)	0.004
**Blood gas analysis indicators**			
PH value	7.35 (7.30, 7.40)	7.39 (7.33, 7.45)	0.011
Lactate levels (mmol/L)	1.7 (1.2, 2.9)	1.4 (1.2, 2.0)	0.041
Blood glucose (mmol/L)	6.6 (5.6, 7.6)	5.2 (4.3, 6.2)	<0.01

*WBC count, white blood cell count; NEUT %, percentage of neutrophils; NE, absolute neutrophil count; LY, absolute lymphocyte count; N/L ratio, neutrophil to lymphocyte ratio; CRP, C-reactive protein; PLT, platelet count; PT, prothrombin time; APTT, activated partial thromboplastin time; FDP, fibrinogen degradation products*.

**Table 3:** ROC values of inflammatory indicators, coagulation function indicators, and blood glucose and lactate level indicators between comparisons of two groups.

**Variables**	**AUC**	***P***	**95%CI**	**Sen**	**Spe**	**Cutoff**
**Inflammatory indicators**						
NEUT%	0.671	0.001	0.577–0.766	0.46	0.84	60.4
N/L ratio	0.646	0.005	0.549–0.742	0.49	0.76	2.03
CRP	0.694	0	0.600–0.789	0.56	0.86	4.18
**Coagulation function indicators**						
D-dimer	0.608	0.039	0.508–0.708	0.36	0.78	2.53
FDP	0.649	0.004	0.552–0.746	0.38	0.92	9.15
**Blood glucose and lactate levels indicators**						
Lactate levels	0.606	0.042	0.506–0.707	0.59	0.61	1.6
Blood glucose	0.703	0	0.608–0.798	0.67	0.76	6.2

*NEUT %, percentage of neutrophils; N/L ratio, neutrophil to lymphocyte ratio; CRP, C-reactive protein; FDP, fibrinogen degradation products; AUC, area under the curve; Sen, sensitivity; Spe, specificity*.

#### The Coagulation Function Indicators

D-dimer and FDP could also be used to predict the disease (NEC with the presence of PVG) among the coagulation function indicators (*P* < 0.05). Although the sensitivities for D-dimer and FDP were similar (0.36 vs. 0.38), the AUC (0.649) and specificity (0.92) of FDP were larger, as shown in Table 3 and Figure 2. No significant differences of PLT, PT, and APTT were found in the study (*P* > 0.05).

#### The Blood Gas Analysis Indicators

Compared to the NEC-non PVG group, lactate levels and blood glucose in the blood gas analysis indicators were both higher in the NEC-PVG group (*P* < 0.05). The median of PH value in the NEC-PVG group was significantly lower than that in the NEC-non PVG group (7.35 vs. 7.39, *P* = 0.011). The AUC (0.703) of blood glucose was higher than those of lactate levels, and we eventually chose it as a better predictive indicator (specificity: 0.67; sensitivity: 0.76), as shown in Table 3 and Figure 2.

### The Surgical Interventions and Outcomes

Sixty-seven neonates reached the criterial of surgical intervention (5) and underwent surgical intervention within 24 h, and their demographic characteristics were reported in Supplementary Table 1. The majority of the surgical interventions in the NEC-non PVG group were laparotomy 48.0%, while the majority of them in the NEC-PVG group were enterostomy, intestinal resection, and anastomosis, which accounted for 90.5% (Table 4).

**Table 4:** Surgical interventions and outcomes in neonates with surgery between both groups.

**Variables**	**NEC-PVG**	**NEC-non PVG**	***P***
	**(*n* = 42)**	**(*n* = 25)**	
**Surgical interventions**			
Laparotomy	4 (9.5%)	12 (48.0%)	0.005
Enterostomy	10 (23.8%)	4 (16.0%)	
Intestinal resection and enterostomy	22 (52.4%)	7 (28.0%)	
Intestinal resection and anastomosis	6 (14.3%)	2 (8.0%)	
**Intraoperative bowel morphology**			
Intestinal perforation	8 (19.0%)	5 (20.0%)	0.924
Intestinal bleeding	15 (35.7%)	9 (36.0%)	0.981
Intestinal necrosis	29 (69.0%)	9 (36.0%)	0.008
**Postoperative complications**			
Intestinal stricture	5 (11.9%)	5 (20.0%)	0.368
Intestinal obstruction	7 (16.7%)	2 (8.0%)	0.525
Short bowel syndrome	3 (7.1%)	1 (4.0%)	0.600
Death	3 (7.1%)	1 (4.0%)	0.600
Subependymal hemorrhage	15 (35.7%)	2 (8.0%)	0.026

The composition ratio of intestinal necrosis was 69.0% in the NEC-PVG group and was just 36.0% in the NEC-non PVG group (*P* < 0.05). Intestinal perforation and bleeding of both groups had no significant differences in the intraoperative bowel morphology (*P* > 0.05). The composition ratio of subependymal hemorrhage in the NEC-PVG group was higher than that in the NEC-non PVG group (35.7% vs. 8%). Three neonates of NEC-PVG group died after surgery, and only one neonate of NEC-non PVG group died.

## Discussion

The incidence rate of NEC, which was 4.99% (718/14,387) in NICU over the 7-year study period, was in accordance with previous reports (2, 3), while the mortality rate was much lower than the reported (0.03%). PVG is associated with more severe NEC and sepsis (19), and it was found to be associated with more acute peritonitis and shock in our study.

In the inflammatory indicators, CRP provided a largest predictive effect than the NEUT% and N/L ratio with the sensitivity of 0.86. Persistently elevated CRP in neonates with NEC suggested associated complications, which required surgical intervention (20). The specificity of CRP levels >4.18 mg/dl for the disease (NEC with the presence of PVG) was 86%, and the sensitivity was 56%. As gram-negative germs produced significantly higher CRP levels than gram-positive germs in VLBW neonates (21), we found that more gram-negative germs (*Klebsiella pneumoniae* and *Escherichia coli*) played an important role in increasing CRP levels. NEUT% provided a second predictive effect with a sensitivity of 0.46 and a specificity of 0.84. A significant increase in NEUT% means that neonates with NEC have serious bacterial infections, and the transcriptional regulator C/EBP-α serves a central function (22). A NEUT% higher than 60.4% probably prompted a significantly increased occurrence of the disease, and it was invalid to only study WBC count. Although Maheshwar (23) found a decrease of NEUT% in severe or late period of NEC, we did not find it in the study because of early examination of laboratory parameters for neonates. de Jager et al. (24) pointed out that the N/L ratio was closely related to the inhibition of the body's immune function; additionally, the absolute lymphocyte counts decreased as disease worsened and the body's immune suppression was aggravated in patients with sepsis. This phenomenon also appeared in our study. When the median of absolute neutrophil counts was similar and the median of absolute lymphocyte counts was lower, there was a significant difference of the N/L ratio between two groups. A 2.03 of N/L ratio was considered as the cutoff value for the disease with a sensitivity of 0.49 and a specificity of 0.76.

Giuliani et al. (13) mentioned that neutrophil elastase, CD63, PROS1, HGF, and F12 were upregulated with an overall procoagulant effect, and MFGE8, factor II (thrombin) receptor-like 1 (F2RL1), FGL2, PLAT, PROCR, SERPIND1, and HNF4A were downregulated with a reduction in fibrinolysis and endothelial regeneration in the early period of NEC. As a result, FDP and D-dimer increased and PT and APTT were prolonged. As diseases deteriorated, D-dimer and FDP increased and disseminated intravascular coagulation (DIC) eventually occurred. Similarly, we found that D-dimer and FDP were significantly increased in the NEC-PVG group. FDP increased above 9.15 mg/L, which was in line with the procoagulant effect, and it was selected as a better predictive indicator on account of high specificity (0.92). High levels of FDP and D-dimer increased the risk of thrombosis and bleeding, and we found a higher occurrence of subependymal hemorrhage after surgery in the NEC-PVG group.

Blood lactate levels reflected inadequate global tissue oxygen delivery rather than a local disease process, and there was a significant difference between neonates who had a necrotic bowel and those who had a healthy bowel (14). Lactate levels >1.6 mmol/L, which was associated with more severe illness, predicted a high occurrence of the disease (NEC with the presence of PVG) (sensitivity: 0.59). García et al. (25) pointed out that neonates requiring surgical intervention presented higher values of glycemia at the diagnosis of NEC, and we also found it in the study. Blood glucose was used as a better predictive indicator with both higher sensitivity of 0.67 and specificity of 0.76. The probable explanation for hyperglycemia appearing more often in the NEC-PVG group was that more severe infectious diseases inhibited the release of insulin, cytokines, or endotoxins, reduced glucose utilization, and increased cortisol or catecholamines.

A loss of integrity of the ischemic and necrotic bowel wall may be the mechanism of PVG (26), which allows intraluminal gas to go through the layers of the wall, translocate into the ischemic tissue, or pass directly into the microvasculature. We found that intestinal necrosis was more likely to appear in the neonates of the NEC-PVG group in the intraoperative bowel morphology. A meta-analysis (27) mentioned that PVG was not associated with surgical intervention or severe complications, and we found that except for subependymal hemorrhage, NEC with the presence of PVG did not increase the occurrence of other outcomes after surgery.

Although this was the first study to put forward the predictive indicators of the disease (NEC with the presence of PVG) in preterm neonates following conservative and surgical interventions, because of the strict selected inclusion applied, only 123 of neonates were included. Additionally, the long-term follow-up throughout childhood is necessary, and we will track it continuously.

## Conclusions

CRP, FDP, and blood glucose had better predictive effects to be used as the predictive indicators for the disease (NEC with the presence of PVG). Intestinal necrosis and subependymal hemorrhage in the outcomes of surgical interventions had a strong relationship with the presence of PVG in NEC II/III. Therefore, early and reasonable use of antibiotics, improvement of coagulation function, rectification of acidosis, and a decrease of blood glucose are suggested.

## Data Availability Statement

The original contributions presented in the study are included in the article/supplementary material, further inquiries can be directed to the corresponding authors.

## Ethics Statement

The studies involving human participants were reviewed and approved by the ethics committee of the Fujian Maternal and Child Health Hospital (Ethics approval number: 2020YJ236). Written informed consent from the participants' legal guardian/next of kin was not required to participate in this study in accordance with the national legislation and the institutional requirements. Written informed consent was not obtained from the individuals, nor the minors' legal guardian/next of kin, for the publication of any potentially identifiable images or data included in this article.

## Author Contributions

XL and H-PZ wrote the main manuscript text. Y-YL prepared Tables 1–4. Y-FF and C-YY amended the manuscript text. All authors reviewed the manuscript.

## Conflict of Interest

The authors declare that the research was conducted in the absence of any commercial or financial relationships that could be construed as a potential conflict of interest.
