# Supplementary material for: Predictive Indicators for Necrotizing Enterocolitis With the Presence of Portal Venous Gas and Outcomes of Surgical Interventions
Source: Front Pediatr. 2021 Jun 14;9:683510. doi: 10.3389/fped.2021.683510 (PMC8238085; doi:10.3389/fped.2021.683510)
Supplement: Supplementary file 1 [file Table_1.docx]

| **Supplementary table 1.** The demographic characteristics of neonates with surgery in the NEC-PVG group and NEC-non PVG group. | | | |
| --- | --- | --- | --- |
| Variables | NEC-PVG | NEC-non PVG | *P* |
|  | (n=42) | (n=25) |  |
| NEC stage (III) | 29(69.0%) | 21(84.0%) | 0.174 |
| GA (week) | 30.0±1.9 | 29.5±2.4 | 0.397 |
| BW (gram) | 1349.4±324.5 | 1371.2±342.5 | 0.795 |
| Male | 26(61.9%) | 17(68.0%) | 0.615 |
| SGA | 8(19.0%) | 1(4.0%) | 0.169 |
| Asphyxia | 7(16.7%) | 3(12.0%) | 0.870 |
| Last volume of milk | 21(15, 27) | 18(15, 29) | 0.599 |
| Mother's age | 29.6±4.3 | 29.8±4.5 | 0.838 |
| Vaginal delivery | 19(45.2%) | 16(64.0%) | 0.137 |
| Premature rupture of membranes | 12(28.6%) | 9(36.0%) | 0.526 |
| Turbid amniotic fluid | 9(21.4%) | 7(28.0%) | 0.542 |
| Multiple pregnancy | 13(31.0%) | 6(24.0%) | 0.541 |
| GDM | 9(21.4%) | 6(24.0%) | 0.807 |
| Hypertension during pregnancy | 8(19.0%) | 3(12.0%) | 0.451 |
| Anemia in pregnancy | 17(40.5%) | 10(40.0%) | 0.969 |
| IAI | 13(31.0%) | 6(24.0%) | 0.541 |
| Abbreviation: NEC, necrotizing enterocolitis; GA, gestational age; BW, birth weight; SGA, small for gestational age; GDM, gestational diabetes mellitus; IAI, intrauterine infection. | | | |
